# Rapamycin reduces neuronal mutant huntingtin aggregation and ameliorates locomotor performance in *Drosophila*

**DOI:** 10.3389/fnagi.2023.1223911

**Published:** 2023-09-26

**Authors:** Jonathan R. Roth, Ruan Carlos Macedo de Moraes, Brittney P. Xu, Savannah R. Crawley, Malghalara A. Khan, Girish C. Melkani

**Affiliations:** ^1^Department of Pathology, Cellular and Molecular Division, Heersink School of Medicine, University of Alabama at Birmingham, Birmingham, AL, United States; ^2^Department of Neurobiology, Heersink School of Medicine, University of Alabama at Birmingham, Birmingham, AL, United States

**Keywords:** Huntington’s disease, aggregation, Htt-polyQ, rapamycin, locomotion, neurodegeneration, *Drosophila*

## Abstract

Huntington’s disease (HD) is a neurodegenerative disease characterized by movement and cognitive dysfunction. HD is caused by a CAG expansion in exon 1 of the *HTT* gene that leads to a polyglutamine (PQ) repeat in the huntingtin protein, which aggregates in the brain and periphery. Previously, we used *Drosophila* models to determine that Htt-PQ aggregation in the heart causes shortened lifespan and cardiac dysfunction that is ameliorated by promoting chaperonin function or reducing oxidative stress. Here, we further study the role of neuronal mutant huntingtin and how it affects peripheral function. We overexpressed normal (*Htt-PQ25*) or expanded mutant (*Htt-PQ72*) exon 1 of huntingtin in *Drosophila* neurons and found that mutant huntingtin caused age-dependent Htt-PQ aggregation in the brain and could cause a loss of synapsin. To determine if this neuronal dysfunction led to peripheral dysfunction, we performed a negative geotaxis assay to measure locomotor performance and found that neuronal mutant huntingtin caused an age-dependent decrease in locomotor performance. Next, we found that rapamycin reduced Htt-PQ aggregation in the brain. These results demonstrate the role of neuronal Htt-PQ in dysfunction in models of HD, suggest that brain-periphery crosstalk could be important to the pathogenesis of HD, and show that rapamycin reduces mutant huntingtin aggregation in the brain.

## 1. Introduction

Huntington’s disease (HD) is a devastating neurodegenerative disease that is characterized by progressive degeneration of the basal ganglia in the brain. This results in a clinical presentation of chorea, which is the spontaneous arrhythmic movement of limbs, alongside cognitive and psychiatric dysfunction. HD is monogenic and is caused by a CAG repeat expansion in exon 1 of the *HTT* gene, leading to a polyglutamine (PQ) expansion in the huntingtin protein. Typically, healthy people have 26 or fewer CAG repeats in *HTT*, while greater than 35 repeats leads to HD, and the age of onset correlates with the CAG repeat number (Duyao et al., 1993; Lee et al., 2012). Currently, there are no effective disease-modifying therapies for HD (Dash and Mestre, 2020).

HD displays prominent dysfunction in both the brain and periphery, especially in muscles. *HTT* is expressed in multiple tissues throughout the body (Strong et al., 1993) and PQ aggregates are present in the brain (Difiglia et al., 1997) and in skeletal and cardiac muscle (Sathasivam et al., 1999), where it can contribute to dysfunction (Van Der Burg et al., 2009). We previously studied the role of mutant huntingtin in the heart because HD patients are at higher risk of dying from cardiac dysfunction (Lanska et al., 1988) and found that mutant huntingtin expressed in the heart causes PQ aggregation, premature mortality, and cardiac dysfunction in *Drosophila* (Melkani et al., 2013). The importance of huntingtin in the periphery is supported by the finding that a mouse model expressing mutant huntingtin ubiquitously has severe cardiac dysfunction (Mihm et al., 2007). Beyond the heart, there is significant evidence that *HTT* is important in skeletal muscle, as movement dysfunction and skeletal muscle weakness are hallmarks of HD (Busse et al., 2008; Zielonka et al., 2014). Additionally, it is possible that brain-muscle crosstalk contributes to dysfunction in HD (Chuang and Demontis, 2021), especially with recent evidence showing that muscle-specific manipulations can affect brain function (Ehlen et al., 2017). However, there is still a gap in knowledge for how brain-muscle crosstalk contributes to dysfunction in HD, and how neuronal *HTT* affects muscle function.

Understanding the mechanisms underlying HD is important for developing effective therapies. *HTT* is involved in axonal trafficking, transcriptional regulation, and cell survival (Schulte and Littleton, 2011). Beyond these important endogenous roles for normal huntingtin, we previously found that oxidative stress and protein misfolding stress contribute to mutant huntingtin–induced dysfunction in the heart (Melkani et al., 2013). Additionally, huntingtin has key roles in regulating autophagy (Cortes and La Spada, 2014), and either huntingtin knockdown or overexpressing mutant huntingtin disrupts autophagosome dynamics (Wong and Holzbaur, 2014). Autophagy dysfunction can contribute to abnormal protein aggregation (Tyedmers et al., 2010), impaired autophagy worsens huntingtin aggregation (Ravikumar et al., 2002), and autophagy is disrupted in models of HD (Martinez-Vicente et al., 2010). Thus, improving autophagy could be beneficial for HD, and it has been proposed to do this with rapamycin (Rubinsztein et al., 2007), which stimulates autophagy by inhibiting TOR (Raught et al., 2001; Li et al., 2014) and improves lifespan in flies and mice (Bjedov et al., 2010). This approach has yielded positive results, as rapamycin reduces mutant huntingtin-associated dysfunction in cell culture and animal models of HD (Ravikumar et al., 2004; Berger et al., 2006; King et al., 2008; Sarkar et al., 2008; Bailus et al., 2021). These studies have focused on the effects of rapamycin on molecular and photoreceptor neuron outcomes, however, and its effects on *in vivo* aggregation and locomotor performance has been understudied in *Drosophila*.

Here, we determined the effects of mutant huntingtin in neurons using well-characterized *Drosophila* models of HD that have proven to be powerful tools (Krench and Littleton, 2013; Lewis and Smith, 2016). We measured the age-dependent effects of neuronal mutant huntingtin on PQ aggregation in the brain and on synapsin levels. We next studied brain-muscle crosstalk to determine whether neuronal mutant huntingtin affects locomotor performance, and if neuron-to-muscle signaling could contribute to dysfunction in the context of HD. Finally, we determined the effects of rapamycin on PQ aggregation in the brain and climbing muscle locomotor performance. Our results determine the age-dependent effects of mutant huntingtin in the brain, support the importance of autophagy in HD, and highlight the potential of rapamycin to prevent aggregation of mutant huntingtin.

## 2. Materials and methods

### 2.1. *Drosophila* stocks and maintenance

As before (Melkani et al., 2013; Gill et al., 2015; Villanueva et al., 2019; Livelo et al., 2023), flies were housed together (about 10 flies per 25 mm vial) in an incubator at 25°C with constant humidity under a 12 h light:12 h dark cycle. Flies were maintained on standard *Drosophila* cornmeal food diet and changed to new food every 4–7 days. We used the following drivers lines: *Elav(X)-Gal4* from Bloomington Drosophila Stock Center (BDSC) (BL#458) and *Elav(II)-Gal4* (BL#8765). We used the following UAS lines: *UAS-eGFP* from BDSC (BL#5431), and HD model lines that expressed huntingtin exon 1 with 72 CAG repeats (*HTTex1-PQ72-GFP*) or 25 CAG repeats (*HTTex1-PQ25-GFP*) tagged with GFP from Dr. Norbert Perrimon (Zhang et al., 2010). Progeny of *Elav* drivers crossed with w^1118^ flies were used as a driver control throughout because the lines were on a w^1118^ background.

### 2.2. Sample preparation for fluorescent imaging

Fly heads were removed under a dissecting microscope and fixed with 4% PFA in PBS for 30 min at room temperature with mild agitation, then washed 3 × 10 min in 1× PBS and stored at 4°C in PBS before cryosectioning. Heads were placed in a mold with OTC and sectioned at 20 μm with a Leica CM3050 S cryostat and placed on glass slides (Fisher #15-188-48). After drying for 30 min, slides were washed 3 × 10 min with 1× PBS to remove dried OTC, then permeabilized with 1× TBST for 10 min. We blocked the slides with 3% BSA in TBST for 30 min and incubated with primary antibody for synapsin (1:500, UI Developmental Studies Hybirdoma Bank #3C11) in 1% BSA in TBST overnight at 4°C in a humidity chamber. Slides were then washed 3 × 10 with PBS and incubated for 1 h at room temperature in AlexaFluor-594 anti-mouse fluorescent secondary antibody (1:1000, ThermoFisher # A-11032). Finally, slides were washed 3 × 10 min with 1× PBS and mounted with ProLong Diamond Antifade Mountant with DAPI (ThermoFisher # P36971). After setting overnight, multichannel fluorescence images were taken with an Olympus BX63F fluorescence microscope using CellSens Software at 10× magnification to view one head section per image.

### 2.3. Htt-PQ-GFP imaging and aggregation quantification

PQ-GFP aggregation was quantified using FIJI software (ImageJ v1.53v) similar to how we have quantified punctate proximity ligation signal before (Rush et al., 2020). The FITC channel images were adjusted in a batch to maximize signal-to-noise, then thresholded to exclude any background signal. ImageJ particle analyzer was run with size and circularity specified to exclude non-punctate signal on a region of interest selected around the brain, and number of puncta per section was reported.

### 2.4. Immunofluorescent imaging synapsin quantification

Syanpsin immunofluorescence intensity was quantified using FIJI software (ImageJ v1.53v) similar to how we have quantified BIN1 protein levels before (Voskobiynyk et al., 2020). The TRITC channel images were adjusted in a batch to maximize signal-to-noise, then mean fluorescent intensity for each image was measured in a region of interest selected around the brain. For each experiment, we ran samples in parallel but with no primary antibody to measure background fluorescence. We measured this background fluorescence in the brain and subtracted mean background fluorescence from the fluorescence value for each image. At least two sections per fly were imaged and mean fluorescence from each fly was calculated and reported.

### 2.5. Negative geotaxis assay

We conducted negative geotaxis assay as before (Ali et al., 2011; Villanueva et al., 2019). Briefly, flies were transferred into clean vials (about 10 per vial) and briefly allowed to acclimate. The vial was sharply tapped onto a pad to stimulate negative geotaxis response, and the proportion of flies the climbed to a 7 cm mark within 10 s was recorded by video for analysis. This was repeated a total of four times for each vial of flies, and the average of the four runs was calculated.

### 2.6. Flight index assay

We conducted flight index assay as before (Drummond et al., 1991; Villanueva et al., 2019). Briefly, one fly at a time was released into a large plexiglass box with a light at the top. Flight index was determined by the fly’s ability to fly up (a flight index value of 6), horizontally (4), down (2), or unable to fly (0). The average flight index was calculated by dividing the sum of individual flight index values by the number of flies for each group.

### 2.7. Rapamycin treatment

Rapamycin (LC Laboratories # R-5000) was dissolved to 50 mM in ethanol, then added to food at 1:250 before pouring into vials for a final concentration of 200 μM, which is sufficient to extend fly lifespan (Bjedov et al., 2010). For vehicle control, ethanol alone was added at 1:250. To avoid developmental effects of rapamycin (Zhang et al., 2000), flies were kept on standard *Drosophila* cornmeal food for 1 week post-eclosion, then split into two groups and put on either rapamycin or vehicle control. Flies were kept on rapamycin or vehicle control for 3 weeks before conducting experiments at 4 weeks of age.

### 2.8. qPCR gene expression

About 10 flies per group per cohort were flash-frozen together and stored at −80°C until use. Heads were removed and put in 150 μL Zymo Lysis Buffer and homogenized at room temperature using the Zymo Quick-RNA MicroPrep Kit (Zymo Research #R1051). RNA was purified using minicolumns and digested with DNAse I for 15 min. Next, the RNA was suspended in RNAse/DNAse free water and quantified with an Aligent Bio Tek Synergy LX Multi-mode and stored at −80°C until use. Next, we used 250 ng of RNA and 4 μL iScript RT Supermax (Bio-Rad #1708840) to synthesize cDNA. Reverse transcription was performed with 5 min of priming at 25°C, 20 min of reverse transcriptase at 46°C, then 1 min of inactivation at 95°C. We used 5 ng of cDNA for qPCR with 200 ng primers per reaction, using Ssa Advanced Universal SYBR Green Supermix (Bio-Rad #1725275) in a CFX Opus Real-Time PCR System (Bio-Rad). Expression was normalized to *Rpl11* (60S ribosomal protein) and *Tbp* (TATA-box binding protein). PCR specificity was verified by comparing the melting curve to a predicted melting curve for the specific sequence generated by the software μMELT Quartz.^1^ Results are presented as 2 − ΔΔCt values normalized to the expression of Rpl11 and Tbp and control samples. All reactions were performed in triplicate.

The following primers were used: *Atg1*-F: CGTCAGCCTGGTCATGGAGTA; *Atg1*-R: TAACGGTATCCTCGCTGAG; *Hid*-F: CACCGACCAAGTGCTATACG; *Hid*-R: GGCGGATACTGGAAGATTTGC; *Pten*-F: GTGCAAACGCAAACAGCCTA; *Pten*-R: ATCCAGTTCTGGTGGCTTCG; *Ilp5*-F: GCGGATTTGGATAGCTCCGA; *Ilp5*-R: AAAGGAACACGATTTGCGGC; *Rpl11*-F: CGATCTGGGCATCAAGTACGA; *Rpl11*-R: TTGCGCTTCCTGTGGTTCAC; *Tbp*-F: ATGCCCTGAGCAACATCCAC; *Tbp*-R: GGATCAGCGGAACCTGGTG.

### 2.9. Statistics

Statistics and test values are described in each figure legend. 1-way or 2-way ANOVA were used, and which effect was tested is reported in each legend along with sample sizes. We used Dunnett’s *post hoc* to compare means to GFP mean. Data values were organized in Microsoft Excel and plotted using Graphpad Prism 9, which was also used for statistical analyses. *α* < 0.05 was defined as our significance threshold. Data were presented as mean ± SEM.

## 3. Results

### 3.1. Neuronal *Htt-PQ72* induces age-dependent aggregation in the brain

**Figure 1:**
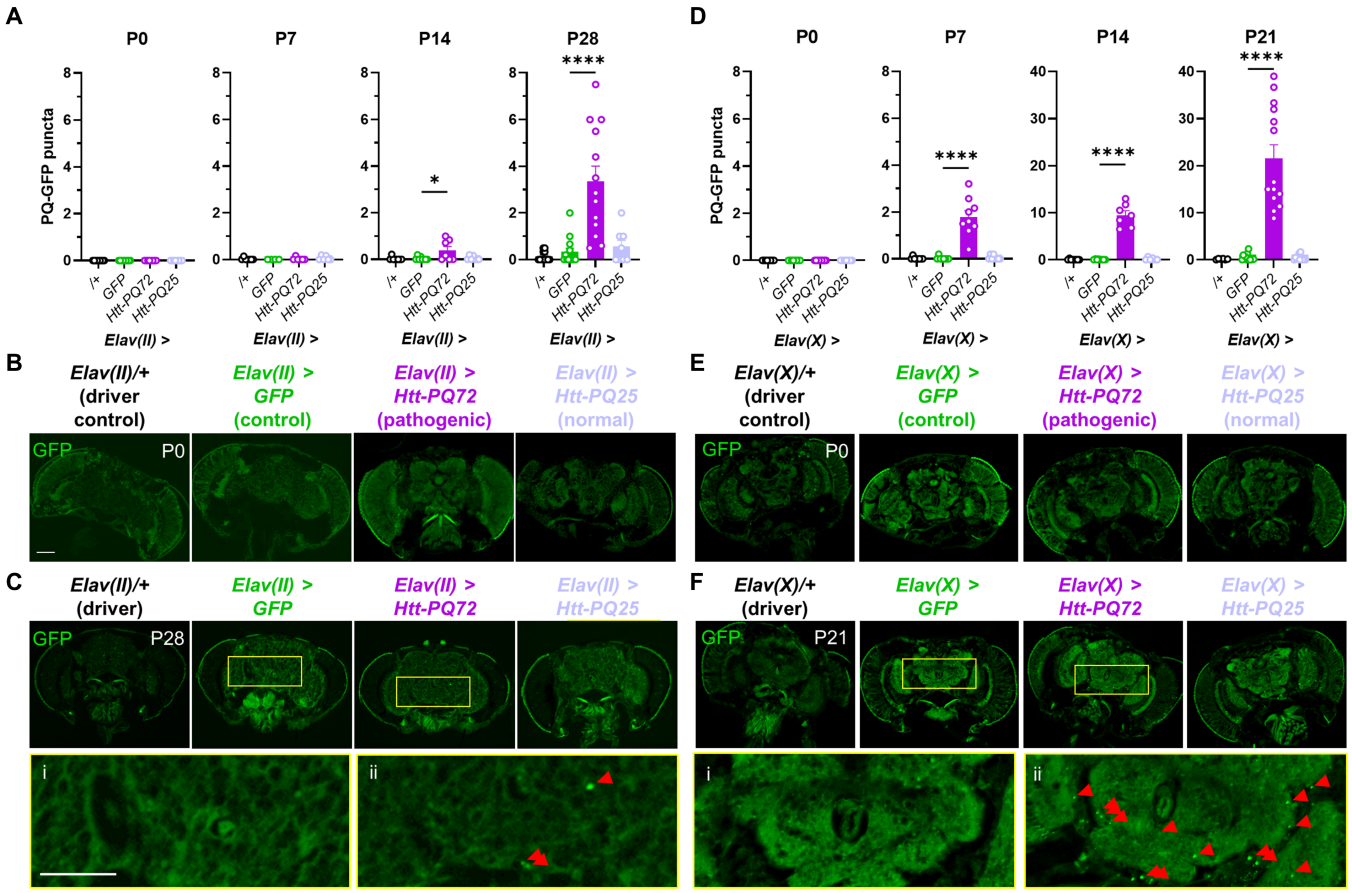
Neuronal *Htt-PQ72* aggregates in the brain. **(A)** Quantification of aggregates in the brain show that PQ-GFP aggregates only in the brains of *Elav(II)* > *Htt-PQ72* flies with age starting at 2 weeks of age (P14), but not in driver control, GFP, or Htt-PQ25 flies (ANOVA for all, P0: *p* = 1.0 because no variability, *N* = 8–9 flies per group; P7: *F*(3,19) = 0.74, *p* = 0.54, *N* = 4–7 flies per group; P14: *F*(3,26) = 4.3, *p* = 0.01, *N* = 7–8 flies per group; P28: *F*(3,42) = 16.6, *p* < 0.0001, *N* = 8–13 flies per group). **(B)** Representative images of flies at eclosion (P0). Scale bar = 100 μm. **(C)** Representative images of heads at 4 weeks of age (P28) showing aggregation. Yellow boxes show inset displayed below of brains of *Elav(II)* > *GFP* flies (i) without aggregates and *Elav(II)* > *Htt-PQ72* flies (ii) with aggregates. Scale bar = 100 μm. **(D)** Quantification of aggregates in the brain show that PQ-GFP aggregates only in the brains of *Elav(X)* > *Htt-PQ72* flies with age starting by 1 week of age (ANOVA for all, P0: *p* = 1.0 because no variability, *N* = 7–9 flies per group; P7: *F*(3,29) = 35.5, *p* < 0.0001, *N* = 8–9 flies per group; P14: *F*(3,25) = 107.5, *p* < 0.0001, *N* = 6–8 flies per group; P21: *F*(3,40) = 37.7, *p* < 0.0001, *N* = 6–14 flies per group). Note the change in *y*-axis between P7 and P14. **(E)** Representative images of heads at eclosion (P0). Scale bar = 100 μm. **(F)** Representative images of heads at 3 weeks of age (P21) showing aggregation. Yellow boxes show inset displayed below of brains of *Elav(X)* > *GFP* flies (i) without aggregates and *Elav(X)* > *Htt-PQ72* flies (ii) with aggregates. Note the higher levels of aggregation when driven by *Elav(X)* compared to *Elav(II)*. All: Data displayed as mean ± SEM. Dunnett’s *post hoc*. *p < 0.05 and ****p < 0.0001 compared to GFP. Red arrows point to PQ-GFP aggregates in the insets.

First, we used the UAS-Gal4 system in *Drosophila* (Osterwalder et al., 2001) to express different huntingtin constructs in neurons. We used the *Elav-Gal4* driver on the 2nd chromosome (*Elav(II)*) to target expression to mature neurons (Robinow and White, 1988), and, along with a driver control crossed with wildtype W^1118^ flies (/+), expressed three UAS constructs in neurons: *GFP* as an expression control, *HTT* exon 1 with 72 CAG repeats fused to GFP (which we refer to as *Htt-PQ72*), and *HTT* exon 1 with 25 CAG repeats fused to GFP (*Htt-PQ25*) (Zhang et al., 2010). We confirmed with fluorescent imaging that the GFP constructs expressed a similar amount (Supplementary Figure S1). While we did not observe any aggregation at eclosion (P0) or 1 week post-eclosion (P7), we found significant PQ-GFP aggregation in the *Elav(II)* > *Htt-PQ72* fly brains at beginning at 2 weeks pose-eclosion (P14) and at 4 weeks post-eclosion (Figures 1A–C). We next verified whether these results were reproducible using a different *Elav-Gal4* line, so we crossed the same UAS constructs with an *Elav-Gal4* driver on the X chromosome (*Elav(X)*). In contrast to when driven by *Elav(II)*, 45% of *Elav(X)* > *Htt-PQ72* died before P28. To avoid survivorship bias, we measured PQ-GFP aggregation at an earlier timepoint at P21. PQ-GFP aggregated much faster in the *Elav(X)* > *Htt-PQ72* flies than the *Elav(II)* > *Htt-PQ72* flies, beginning by P7, and having much higher aggregation by 3 weeks (P21) than then *Elav(II)* > *Htt-PQ72* flies had at P28 (Figures 1D–F). We interpreted that this could be due to higher expression with the *Elav(X)* driver than with the *Elav(II)* driver, which could also explain the differences in premature mortality between the drivers. Together, these results show that overexpressing mutant huntingtin in neurons causes an age- and driver-dependent aggregation of PQ-GFP in the brain.

### 3.2. Neuronal *Htt-PQ72* can induce synapsin loss in the brain

**Figure 2:**
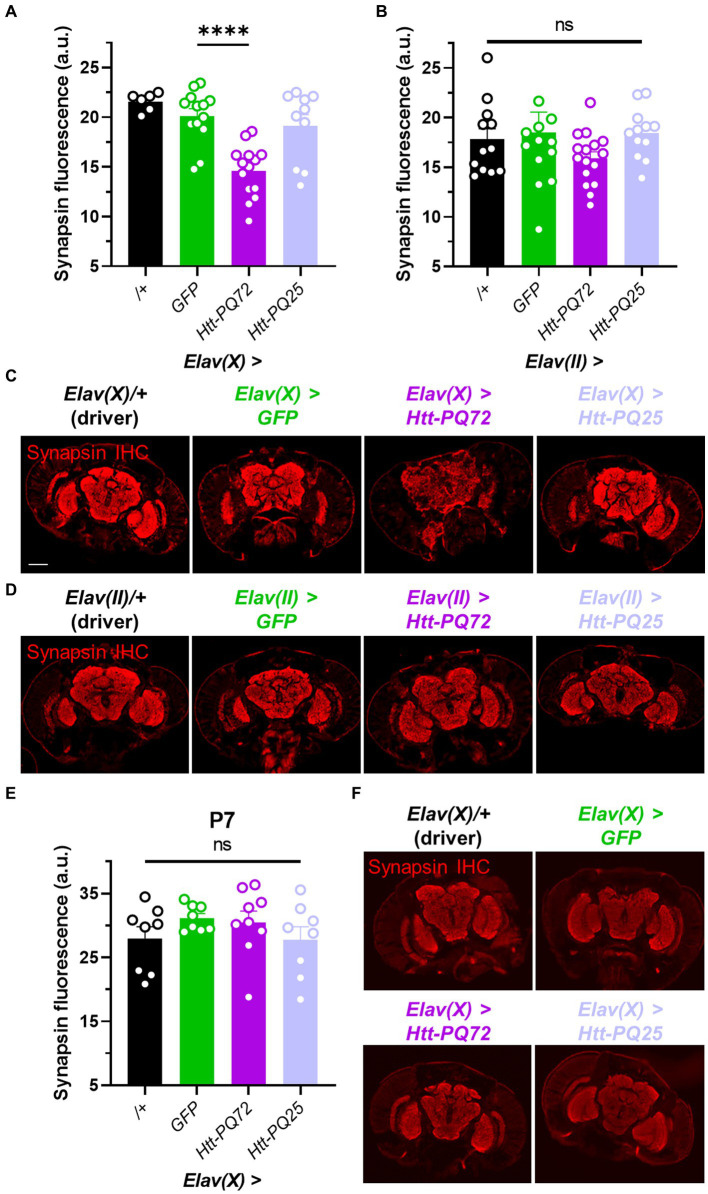
Neuronal *Htt-PQ72* can induce synapsin loss in the brain. **(A)** Quantification of synapsin staining shows a decrease of synapsin in *Elav(X)* > *Htt-PQ72* flies at P21 (ANOVA *F*(3,39) = 13.6, *p* < 0.0001, Dunnett’s *post hoc* ****p < 0.0001 compared to *GFP*, *N* = 6–14 flies per group). **(B)** Quantification of synapsin staining shows no differences in synapsin levels in the brains of flies driven by *Elav(II)* at P28 (ANOVA *F*(3,49) = 1.1, *p* = 0.37, *N* = 12–16 flies per group). **(C)** Representative immunofluorescent images stained for presynaptic marker synapsin in the brain of P21 flies driven by *Elav(X)*. Scale bar = 100 μm. **(D)** Representative images of P28 *Elav(II)* fly heads stained for synapsin. **(E)** Quantification of synapsin staining shows no difference between any groups driven by *Elav(X)* at P7, when aggregates start to form (ANOVA *F*(3,29) = 1.0, *p* = 0.39, *N* = 8–9 flies per group). **(F)** Representative images of P7 *Elav(X)* fly heads stained for synapsin. All: Data displayed as mean ± SEM.

Synaptic dysfunction is a common early feature in neurodegeneration, especially HD (Smith-Dijak et al., 2019), and synapse loss often precedes neuronal loss (Milnerwood and Raymond, 2010). Thus, we used immunohistochemistry to measure synapsin levels to determine whether *Htt-PQ72* could induce changes at the synapse that are consistent with synaptic dysfunction. Synapsin is critical for neurotransmitter vesicle release (Cesca et al., 2010) and binds mutant huntingtin in the presynaptic compartment to inhibit its phosphorylation (Xu et al., 2013), which predicts dysfunction in HD models (Liévens et al., 2002). We found that at 3 weeks of age, *Elav(X)* > *Htt-PQ72* flies had reduced synapsin levels in the brain relative to *GFP*, while *Elav(X)* > *Htt-PQ25* flies did not (Figures 2A,C). However, we did not see a significant change in synapsin staining in any flies using the *Elav(II)* driver at 4 weeks of age (Figures 2B,D). To determine whether synapsin loss occurs with PQ-GFP aggregation or after significant aggregation, we stained for synapsin in P7 *Elav(X)* flies, when PQ-GFP aggregation has already occurred (Figure 1A), and found that there was no difference in synapsin staining (Figures 2E,F). Thus, PQ-GFP aggregation precedes synapsin loss in the brain of *Elav(X)* > *Htt-PQ72* flies, which occurs with age.

### 3.3. Neuronal *Htt-PQ72* causes age-dependent dysfunction in peripheral locomotor dysfunction

**Figure 3:**
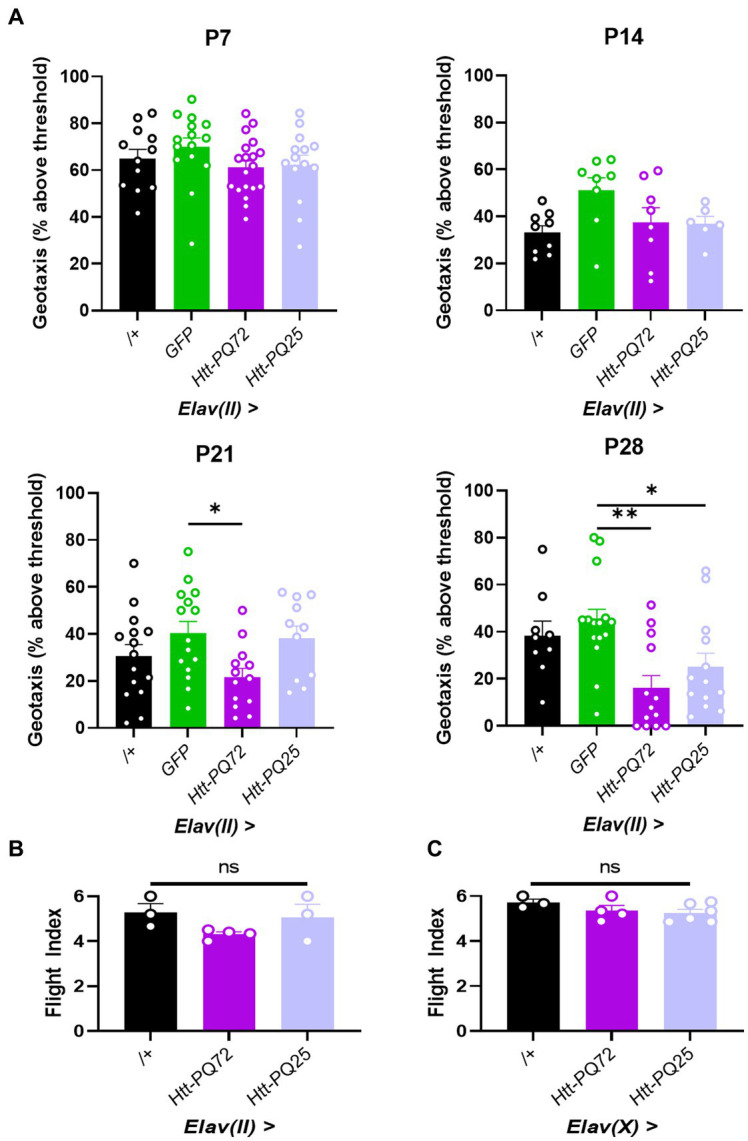
Neuronal *Htt-PQ72* decreases climbing muscle performance with age. **(A)** Negative geotaxis assay demonstrates progressive loss of climbing muscle performance in *Elav(II)* > *Htt-PQ72* flies beginning at P21 (ANOVA for all, P7: *F*(3,56) = 1.2, *p* = 0.31, *n* = 12–19 cohorts per group; P14: *F*(3,27) = 2.9, *p* = 0.06, *n* = 6–9 cohorts per group; P21: *F*(3,50) = 3.2, *p* = 0.03, *n* = 11–15 cohorts per group; P28: *F*(3,46) = 5.6, *p* = 0.002, *n* = 9–15 cohorts per group. All: Dunnett’s *post hoc* *p < 0.05 and **p < 0.01 compared to GFP). **(B)** Flight index assay demonstrates no significant difference in flight muscle performance in *Elav(II)*-driven flies at P28 (ANOVA *F*(2,7) = 2.2, *p* = 0.19, *n* = 3–4 cohorts with *N* = 14–19 flies per group). **(C)** Flight index assay demonstrates no significant difference in flight muscle performance in *Elav(X)*-driven flies at P21 (ANOVA *F*(2,10) = 1.5, *p* = 0.29, *n* = 3–5 cohorts with *N* = 11–35 flies per group). All: Data displayed as mean ± SEM.

HD is characterized by muscle dysfunction, and we have previously found that *Htt-PQ72* causes dysfunction in cardiac and skeletal muscle (Melkani et al., 2013; Barwell et al., 2023). It has been proposed that this peripheral muscle dysfunction is primarily caused by peripheral expression of mutant huntingtin, and that peripheral mutant huntingtin could cause neuronal dysfunction in the brain (Chuang and Demontis, 2021). We wanted to complement this idea and test the converse – whether neuronal huntingtin can disrupt locomotor performance independent from expression in the muscle. To do so, we conducted two assays to measure the performance of two types of skeletal muscle: negative geotaxis assay, which measures climbing muscle performance, and flight index assay, which measures flight muscle performance (Villanueva et al., 2019). We chose to use the *Elav(II)* driver to allow for observing more subtle phenotypes induced by lower aggregation or expression of constructs. While there were no significant changes in climbing muscle performance at P7, before PQ-GFP aggregation, or at P14, when aggregation is beginning (Figure 1A), *Elav(II)* > *Htt-PQ72* flies displayed progressive dysfunction in climbing muscle performance starting at P21, beyond the loss of muscle performance associated with normal aging (Figure 3A). There was also some climbing muscle dysfunction in *Elav(II)* > *Htt-PQ25* flies at P28, which is not surprising since some dysfunction has been reported by overexpression of shorter PQ chains with aging *in vivo* (Morley et al., 2002). To determine if neuronal mutant huntingtin causes general muscle performance loss, we performed a flight index assay to measure flight muscle performance. To our surprise, we found no change in flight muscle performance at P28 (Figure 3B). As it is possible that higher PQ-GFP in neurons could decrease flight muscle performance, we repeated the flight index assay using the *Elav(X)* driver. Again, we observed no change in flight muscle performance at 3 weeks of age (Figure 3C). Together, these results show that neuronal *Htt-PQ72* can decrease climbing muscle performance in an age-dependent manner without decreasing flight muscle performance.

### 3.4. Rapamycin treatment reduces *Htt-PQ72* aggregation in the brain and ameliorates locomotor performance

**Figure 4:**
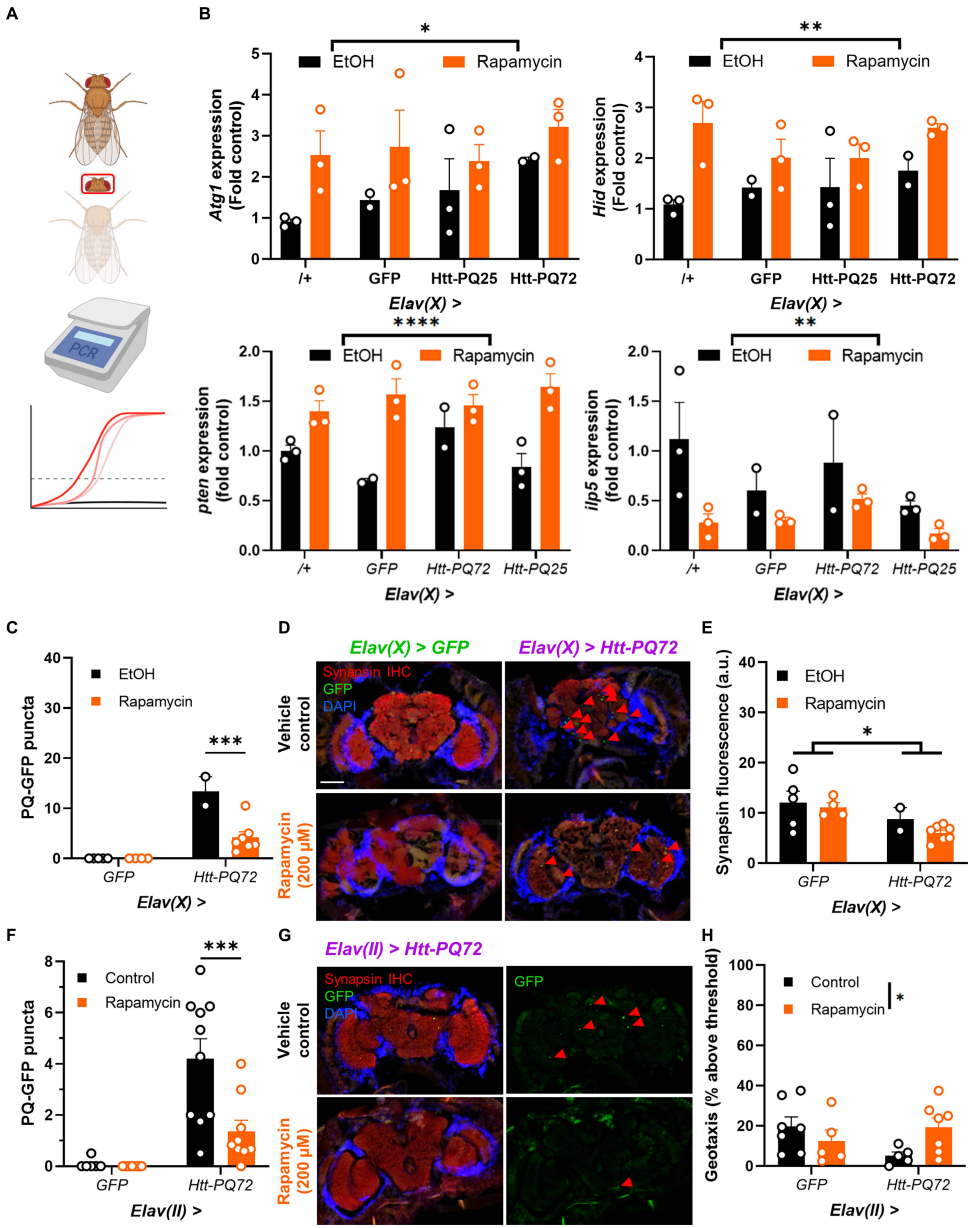
Rapamycin reduces *Htt-PQ72* aggregation in the brain and ameliorates flight muscle performance. **(A)** qPCR workflow. Fly heads were isolated for RNA extraction and qPCR was used to compare transcript levels between groups. This image was created in Biorender. **(B)** Rapamycin increases *Atg1* expression (*F*(1,14) = 7.218, *p* = 0.0173), *Hid* expression (*F*(1,14) = 13.25, *p* = 0.0027), and *pten* expression (2-way ANOVA main effect of rapamycin *F*(1, 14) = 39.7, *p* < 0.0001); and decreases *ilp5* expression (2-way ANOVA main effect of rapamycin *F*(1, 14) = 10.0, *p* = 0.007). *N* = 2–3 cohorts with *n* = about 10 flies per group per cohort of fly heads. **(C)** Quantification of PQ-GFP aggregates in *Elav(X)*-driven flies treated with 200 μM rapamycin or vehicle control for 3 weeks. Rapamycin decreases aggregation in *Elav(X)* > *Htt-PQ72* fly brains (2-way ANOVA interaction *F*(1,14) = 15.26, *p* = 0.002, Dunnett’s *post hoc* ***p < 0.001 compared to vehicle control, *N* = 2–7 flies per group). **(D)** Representative fluorescent images of P28 *Elav(X)*-driven GFP and Htt-PQ72 flies with vehicle control or Rapamycin for 3 weeks. Scale bar = 100 μm. **(E)** Quantification of synapsin staining shows a decrease of synapsin in *Elav(X)* > *Htt-PQ72* flies at with no effect of rapamycin (2-way ANOVA main effect of genotype *F*(1,14) = 6.4, *p* = 0.02, *N* = 2–7 flies per group). **(F)** Quantification of PQ-GFP aggregates in *Elav(II)*-driven flies treated with 200 μM rapamycin or vehicle control for 3 weeks. Rapamycin decreases aggregation in *Elav(II)* > *Htt-PQ72* fly brains (2-way ANOVA interaction *F*(1,30) = 6.9, *p* = 0.014, Dunnett’s *post hoc* ***p < 0.001 compared to vehicle control, *N* = 6–10 flies per group). **(G)** Representative fluorescent images of P28 *Elav(II)* > *Htt-PQ72* flies treated with vehicle control or rapamycin for 3 weeks. **(H)** Rapamycin modifies the loss of climbing performance in *Elav(II)* > *Htt-PQ72* flies at P28 (2-way ANOVA interaction *F*(1,20) = 5.2, *p* = 0.03, *n* = 5–7 cohorts with *N* = 34–73 flies per group).

Autophagy plays an important role in aging and metabolism, and has been implicated in many neurodegenerative diseases including HD (Croce and Yamamoto, 2019). Rapamycin stimulates autophagy and prevents some mutant huntingtin-induced dysfunction (Djajadikerta et al., 2020), so we next tested whether it impacted PQ-GFP aggregation in the brain. We placed 1 week-old flies on food supplemented with 200 μM rapamycin or vehicle control food for 3 weeks. First, we verified the mechanism of action of rapamycin by isolating heads and conducting PCR for genes associated with autophagy and TOR signaling (Figure 4A). We found that rapamycin significantly increases expression of *Atg1*, which is responsible for the formation of autophagosomes (Mizushima, 2010; Stjepanovic et al., 2014). Additionally, we measured expression of *Hid*, which can induce autophagy (Baehrecke, 2003; Juhász and Sass, 2005), and *pten*, which is negatively associated with TOR activity (Manning and Cantley, 2007; Das et al., 2012), and found them increased by rapamycin, demonstrating that rapamycin treatment led to inhibition of TOR as expected. As an independent measure of TOR activity, we measured *ilp5* expression, which is positively regulated by TOR and is important for nutrient signaling and metabolism (Texada et al., 2022; Ling and Raikhel, 2023). Rapamycin significantly decreased *ilp5*, again demonstrating that rapamycin inhibited TOR signaling (Figure 4B). Next, we determined whether rapamycin prevented PQ-GFP aggregation and other mHTT-associated dysfunction. We found that rapamycin reduced PQ-GFP aggregation, though it did not prevent a loss of synapsin in aged *Elav(X)* flies (Figures 4C–E). Next, we determined whether rapamycin was sufficient to prevent PQ-GFP aggregation and locomotor performance loss in *Elav(II)* flies. We found that rapamycin again reduced PQ-GFP aggregation and also prevented a loss of locomotor performance in *Elav(II)* > *Htt-PQ72* flies (Figures 4F–H). There was a decrease in climbing performance in the control flies relative to prior experiments (Figure 3), likely due to chronic ethanol exposure (Chvilicek et al., 2020; Nuñez et al., 2023) as ethanol was used to dissolve the rapamycin and was included in the control food as a vehicle. Altogether, these results show that rapamycin is sufficient to prevent aggregation of mutant huntingtin in the brains of flies and can prevent the loss of locomotor performance.

## 4. Discussion

In this work, we describe the effect of expressing *Htt-PQ72* in *Drosophila* neurons. We characterized age-dependent aggregation of PQ-GFP that was greater when driven by *Elav(X)* than *Elav(II)*, and that it can reduce synapsin in the brain after PQ-GFP aggregation occurs. We next explored the role of neuronal *Htt-PQ72* in walking muscle performance and found that it causes age-dependent loss of climbing muscle performance but no effect on flight muscle performance. Finally, we found that rapamycin reduces aggregation of PQ-GFP in the brain and prevents the loss of locomotor performance, supporting the importance of autophagy in HD models and that rapamycin could be beneficial to prevent mutant huntingtin–induced dysfunction.

Brain-periphery crosstalk is likely important in many neurodegenerative diseases, especially HD. Huntingtin is expressed in many tissues throughout the body and mutant huntingtin aggregates have been found in multiple tissues (Van Der Burg et al., 2009). Most preclinical work in HD has studied the role of neuronal huntingtin in the brain and peripheral huntingtin in periphery, though new studies in brain-periphery crosstalk have demonstrated that the periphery can affect the brain, especially skeletal muscles (Ehlen et al., 2017; Matthews et al., 2022). Our results complement these studies and add to the evidence supporting the importance of brain-periphery crosstalk, as neuronal mutant huntingtin is sufficient to reduce locomotor performance without expression of mutant huntingtin in the muscles themselves.

We found it interesting that *Htt-PQ72* caused a loss of climbing muscle performance but not flight muscle performance. This suggests that there are specific effects of neuronal mutant huntingtin on specific peripheral tissues, and highlights the importance of studying multiple types of skeletal muscle in models of movement disorders like HD. One potential caveat to these results is that the negative geotaxis assay is dependent on neuronal function (Sun et al., 2018), so it is possible that the climbing muscles themselves were not disrupted, *per se*, but were impacted by neuronal dysfunction caused by *Htt-PQ72*. This is supported by the lack of phenotype in the flight muscle performance assay, which is not primarily driven by neuronal function. Additionally, *Elav* is expressed in peripheral neurons in addition to central nervous system neurons (Robinow and White, 1988), so it is possible that the locomotor dysfunction is due to peripheral neuron dysfunction outside of the brain. Alternatively, it is possible that mHTT toxicity in areas of the brain that control movement could contribute to the locomotor dysfunction, as is hypothesized to occur in humans (Smith et al., 2000). Altogether, it is notable that neuronal mutant huntingtin is sufficient to specifically reduce skeletal muscle performance, likely due to neuronal dysfunction, especially with the prominence of locomotor dysfunction present in HD.

Another potential caveat to our results is the differences seen with using the *Elav(II)* and *Elav(X)* drivers, especially regarding the synapsin loss (Figure 2). Two potential explanations for the differences are that they are due to either differences in expression location or different expression levels. Both driver lines are well-established models to express constructs in mature neurons that endogenously express *Elav*, and there is typically minimal leakage with the UAS-Gal4 system (Osterwalder et al., 2001). Our results were consistent with differences being due to higher expression with the *Elav(X)* driver, namely the early mortality, higher level of aggregation at an earlier age, and the reduced synapsin. It is notable that PQ-GFP aggregation in P7 *Elav(X)* > *Htt-PQ72* flies and P28 *Elav(II)* > *Htt-PQ72* flies (1.78 vs. 3.36 aggregates per brain section) was closer than differences in aggregation between P21 *Elav(X)* > *Htt-PQ72* flies and P28 *Elav(II)* > *Htt-PQ72* flies (21.57 vs. 3.36 aggregates per brain section), so we speculate that it is possible that a reduction of synapsin levels would occur in the *Elav(II)* > *Htt-PQ72* flies with more age and PQ-GFP aggregation. Thus, we expect that the differences were due to *Elav(X)* driving higher expression than *Elav(II)*, though we cannot exclude the possibility of different cell-specific expression of the drivers or Gal4 leakage.

The difference between models is also seen in effects of rapamycin, which reduces PQ-GFP aggregation in both models and prevents the loss of locomotor performance in *Elav(II)* > *Htt-PQ72* flies, but does not prevent the loss of synapsin in *Elav(X)* > *Htt-PQ72* flies (Figure 4). There are several possible explanations for this. It is possible that the more severe model (*Elav(X)*) had too much mHTT-associated toxicity for this dose of rapamycin to prevent synapsin loss, that treating with rapamycin after aggregation occurred was insufficient to prevent it, or that synapsin loss is a downstream effect of mHTT that is independent of its effects of aggregation and locomotor performance. We speculate that it is due to a ceiling effect such that the *Elav(X)* model has high enough expression that mHTT toxicity has either already occurred or that the dose of rapamycin used was not sufficient to prevent mHTT toxicity.

Both PQ-GFP aggregation and synapsin loss were spread uniformly throughout the brain. This was expected, as *Elav-Gal4* drives expression pan-neuronally in all parts of the brain, so *Elav* > *Htt-PQ72* will be expressed in all brain regions and can aggregate throughout. Additionally, since soluble mHTT can be toxic (Leitman et al., 2013), aggregation is not necessary for downstream dysfunction, as is seen with synapsin loss (Figure 2). Altogether, our results support the idea that high levels of non-aggregated mHTT can cause neuronal dysfunction in multiple neuron subtypes throughout the brain, namely a decrease in synapsin.

Currently, there are no highly effective treatments for HD. Rapamycin has been proposed as a potential therapeutic strategy because mutant huntingtin impairs autophagy, leading to neuronal dysfunction (Pircs et al., 2018). Multiple preclinical studies have demonstrated that rapamycin can delay aging (Harrison et al., 2009; Bjedov et al., 2010; Robida-Stubbs et al., 2012), even after just brief treatment during adulthood (Juricic et al., 2022), and that it prevents mutant huntingtin-induced dysfunction (Ravikumar et al., 2004; Sarkar et al., 2008). Our data support this approach as we found that rapamycin reduces mutant huntingtin–induced dysfunction in the brain and periphery. It is important to note that rapamycin can inhibit huntingtin aggregation independent of autophagy (King et al., 2008), and that aggregation may in fact be protective by reducing toxic, soluble forms of mutant huntingtin (Arrasate et al., 2004). Thus, it is important that we found that rapamycin prevented reduced behavioral dysfunction, especially progressive muscle performance dysfunction caused by neuronal mutant huntingtin. HD is rare within neurodegenerative diseases in that it is autosomal dominant and monogenic (Myers, 2004). Thus, it could be possible to identify individuals who will develop HD and treat them before symptom onset if rapamycin is found to prevent development of motor dysfunction in HD. Interestingly, HD is part of a class of neurodegenerative diseases that are characterized by PQ repeat expansions, including several ataxias (Shao and Diamond, 2007), so we speculate that rapamycin could be beneficial in these diseases if there are common mechanisms underlying PQ expansion disorders.

In summary, this study explores the role of mutant huntingtin in neurons. It shows that expressing mutant huntingtin in *Drosophila* neurons causes an age-dependent increase in mutant huntingtin aggregation in the brain, which can be associated with synapsin loss. Additionally, it supports the importance of brain-muscle crosstalk in HD models, as we found age-dependent loss of climbing muscle performance caused by mutant huntingtin in neurons. Finally, this study adds to the body of work showing the importance of autophagy in HD and the potential therapeutic benefits of rapamycin in HD models.

## Data availability statement

The original contributions presented in the study are included in the article/Supplementary material, further inquiries can be directed to the corresponding author.

## Ethics statement

The manuscript presents research on animals that do not require ethical approval for their study.

## Author contributions

JRR, RCM, and GCM contributed to the initial study and experimental design. JRR, RCM, BPX, SRC, and MAK conducted experiments, analysed, and interpreted results. JRR wrote the manuscript with input from RCM, BPX, SRC, MAK, and GCM. All authors contributed to the article and approved the submitted version.

## Footnotes

^1^ www.dna-utah.org/umelt/quartz/
