# Supplementary material for: Rapamycin reduces neuronal mutant huntingtin aggregation and ameliorates locomotor performance in Drosophila
Source: Front Aging Neurosci. 2023 Sep 26;15:1223911. doi: 10.3389/fnagi.2023.1223911 (PMC10562706; doi:10.3389/fnagi.2023.1223911)
Supplement: Supplementary file 1 [file Presentation_1.pdf]

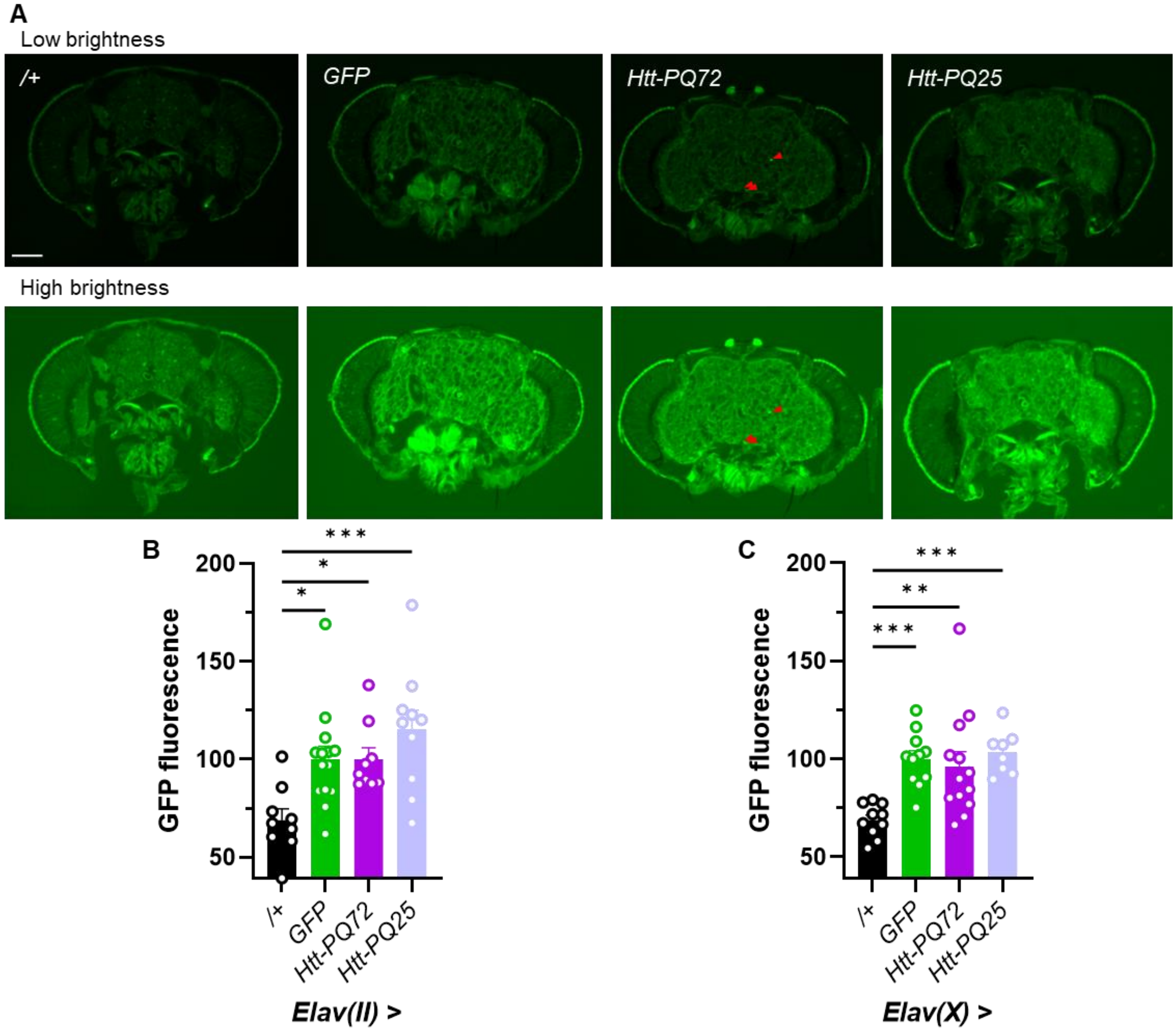

Supplementary Figure 1: *Elav* drives similar GFP expression in *GFP, Htt-PQ72,* and *Htt-PQ25* fly brains. (A) Representative images of P28 *Elav(II)* fly heads. Top row is low brightness, which shows aggregates in *Elav(II) > Htt-PQ72* brains. Red arrows point to PQ-GFP aggregates in the insets. These are the same images in Figure 1C. Bottom row is brightened to show GFP expression in the brain of flies expressing *GFP*, *Htt-PQ72*, and *Htt-PQ25*, but not driver control, though it is now hard to see aggregates (red arrows). Scale bar = 100 µm. **(B)** Quantification of GFP fluorescence shows significant expression of GFP in *GFP*, *Htt-PQ72*, and *Htt-PQ25* compared to background fluorescence in driver control flies driven by *Elav(II)* at P28 (ANOVA $F(3,38) = 6.1$, $p = 0.002$, Dunnett's *post hoc* *: $p = 0.01$, ***: $p < 0.001$ compared to driver control, N = 8–13 flies per group). **(C)** Quantification of GFP fluorescence shows significant expression of GFP in *GFP*, *Htt-PQ72*, and *Htt-PQ25* compared to background fluorescence in driver control flies driven by *Elav(X)* at P21 (ANOVA $F(3,38) = 7.7$, $p = 0.004$, Dunnett's *post hoc* **: $p < 0.01$, ***: $p < 0.001$ compared to driver control, N = 8–13 flies per group).
